# Social Prescribing Schemes in Primary Care in Spain (EvalRA Project): a mixed-method study protocol to build an evaluation model

**DOI:** 10.1186/s12875-023-02164-9

**Published:** 2023-10-25

**Authors:** M. Pola-Garcia, A. M. Carrera Noguero, M. P. Astier-Peña, J. J. Mira, M. Guilabert-Mora, V. Cassetti, E. Melús-Palazón, A. Gasch-Gallén, Consortium EvaLRA, N. Enríquez Martín, A. Asencio Aznar, C. Gimeno-Monterde, K. Cheikh-Moussa, M. L. Lou Alcaine, C. B. Benedé Azagra

**Affiliations:** 1 Servicio Aragonés de Salud, Zaragoza, Spain; 2 Grupo GIIS011, Instituto de Investigación Sanitaria de Aragón, Zaragoza, Spain; 3 Programa Actividades Comunitarias en Atención Primaria (PACAP), Sociedad Española de Medicina Familiar y Comunitaria (SEMFYC), Barcelona, Spain; 4 Unidad Territorial de Calidad, Dirección Territorial del Camp de Tarragona, Institut Català De La Salut, Tarragona, Spain; 5 FEPS, Instituto de Investigación Sanitaria de Aragón, Zaragoza, Spain; 6 Wonca World Executive Board, Brussels, Belgium; 7 Grupo de trabajo de Seguridad del Paciente, Sociedad Española de Medicina Familiar y Comunitaria (SEMFYC), Barcelona, Spain; 8 Departmento Psicología de la Salud, Universidad Miguel Hernandez, Alicante, Spain; 9 Grupo de Investigación Atenea, Fundación para la Investigación Biomédica de la Comunidad Valenciana (FISABIO), Alicante, Spain; 10 Calité Investigación, Universidad Miguel Hernandez, Alicante, Spain; 11 Departamento de Salud Alicante-San Juan de Alicante, Alicante, Spain; 12 Universidad Internacional de Valencia (VIU), Valencia, Spain; 13 Instituto de Investigación Sanitaria de Aragón, Zaragoza, Spain; 14 Indepent research, Affiliated researcher to the Unesco Chair in Global Health and Education, London, UK; 15 Grupo Aragonés de Investigación en Atención Primaria B21_23R, Gobierno de Aragón, Zaragoza, Spain; 16 Departamento de Medicina, Psiquiatría y Dermatología, Universidad de Zaragoza, Zaragoza, Spain; 17 Departamento de Fisiatria y Enfermería, Universidad de Zaragoza, Zaragoza, Spain; 18 Grupo GIIS094, Instituto de Investigación Sanitaria de Aragón, Zaragoza, Spain; 19 Estrategia de Atencion Comunitaria en el Sistema de Salud de Aragon Atencion Primaria. Servicio Aragones de Salud, Departamento de Sanidad, Gobierno de Aragon, Zaragoza, Spain

**Keywords:** Evaluation studies, Social prescribing, Primary health care, Community health services, Community networks

## Abstract

**Background:**

Social Prescribing is a Primary Health Care service that provides people with non-clinical care alternatives that may have an impact on their health. Social Prescribing can be more or less formal and structured.

Social Prescribing Schemes are formal Social Prescribing of health assets by Primary Health Care teams in coordination and follow-up of patients with providers.

The emerging evidence suggests that this service can improve people’s health and well-being, create value and provide sustainability for the healthcare system. However, some evaluations note that the current evidence regarding social prescribing is insufficient and needs further investigation.

The EvaLRA project aims to elaborate an evaluation model of Social Prescribing Schemes in Primary Health Care based on a set of structure, process, and outcomes indicators.

**Methods:**

In the region of Aragon, the Community Health Care Strategy aims to promote the development of social prescription schemes in Primary Health Care teams.

This study is divided into two stages. Stage 1: identification of primary health care teams that implement social prescribing schemes and establish a first set of indicators to evaluate social prescribing using qualitative consensus techniques with experts. Stage 2 evaluation of the relevance, feasibility and sensitivity of selected indicators after 6 and 12 months in primary health care teams. The results will provide a set of indicators considering structure, process and outcomes for social prescribing schemes.

**Discussion:**

Current evaluations of the application of social prescribing schemes use different criteria and indicators. A set of agreed indicators and its piloting in primary health care teams will provide a tool to evaluate the implementation of social prescription schemes. In addition, the scorecard created could be of interest to other health systems in order to assess the service and improve its information system, deployment and safety.

## Background

In Spain, community health care is a basic public primary health care service (PHC) offered in all the national health system [1]. It includes actions aimed at detecting and prioritizing the health needs and problems of people in an area, identifying available community resources and creating programs to improve the community health. This service involves the participation of the community and the collaboration with other sectors [1].

The service focuses on positive health which involves two important aspects, salutogenesis and health assets [2, 3].

Salutogenesis puts the emphasis on the issues which generate health and develop procedures in order to identify and measure the factors that cause a better understanding of health and quality of life for people [4].

The health assets, defined by Morgan and Ziglio in 2007 are any factor or resource which enhances the ability of individuals, communities and populations to maintain and sustain health and well-being [2].

Adopting a health assets approach to community health care encourages cross-sectoral cooperation, community involving to address equity and social determinants of health. This approach builds up a health improving processes among individuals, communities and health care and social care professionals in a particular context. Health assets can act at the individual, family or community level [2, 5]. When the health assets are prescribed by PHC care professionals, it is called Social Prescribing (SP). There are different models of social prescription [6]. SP is a tool in PHC Health Care that provides people with non-clinical care alternatives that have an impact on their health [7].

In 2015, Kimberlee established 4 levels of social prescribing and organized them according to their structure and coordination with different community resources [8]. Considering Kimberlee classification, first and second levels refer to the recommendation of community resources or activities within a therapeutic framework without formal coordination between PHC teams and the health asset providers in the community. Therefore, with lack of monitoring and evaluation (named as "non formal social prescribing"). Levels three and four refer to the creation of formal social prescribing schemes in which there is a coordination set up between PHC teams and the health assets providers with a monitoring and evaluation process (named as "formal social prescribing") [8, 9].

Currently, healthcare services have a growing interest in developing SP in a more organized and formal way in PHC (Kimberlee levels three and four) due to several factors. Some of them are the growing demand for health care services, the ageing of the population, the prevalence of chronic diseases, the growth in pharmaceutical prescription or the need for a joint approach to the social determinants of health among others [10–13].

Implementing SP provides benefits in terms of emotional well-being, empowerment, sociability, communication skills or the improvement of personal social networks. Moreover, the results of some studies have suggested that patients are satisfied with the formal social prescribing frameworks. However, some evaluations note that the current evidence regarding SP is insufficient and that there needs to be further research on not only the possible beneficiaries of social prescribing frameworks but also on the health outcomes of social prescribing and the cost-effectiveness of interventions [14–20].

There are several countries which implement social prescribing programs [21, 22]. Those interventions involve not only the healthcare systems but the social system and other agents as well. Therefore, they are customized interventions adapted to the particular settings and which are difficult to assess and being accountable to governments and society [22].

In Spain, the 2019 National Strategic Framework for Primary and Community Health Care aims to develop SPS and tools, which will include medium-term monitoring and evaluating mechanisms [23]. Likewise, different initiatives have been developed in some of the regional health services across Spain as in Aragon region [24].

However, few evaluation frameworks have been established. Upon reviewing the literature, we found small-scale and short-term evaluations and a powerful model for assessment of SP focused in results. Nevertheless, there is not a set of basic criteria and indicators to assess SP services [19, 25–27].

Thus, evaluating SPS is a challenge due to the fact that actors from different administrations are involved as well as the lack of information systems which allow interoperability between them. SPS address many different health issues through a large variety of interventions. Moreover, many social determinants have an influence on health, and changes may be visible on a long-term basis. The present study aims to answer the question of providing an evaluation for SPS considering the perspective of healthcare systems including patient, professional and provider’s satisfaction as well. Therefore, “EvaLRA: The development of a model of evaluation indicators in Social Prescribing Schemes in Primary Health Care” [28], aims to develop a set of indicators for SPS in PHC teams in Aragon Healthcare System (Spain), which may be useful at an international level.

## Methods

The evaluation is going to be developed in PHC teams of a Spanish health region with an established Community Health Strategy and an electronic health record (EHR) information system that includes SPS protocol for a formal implementation.

Aragon is a region in the northeast of Spain whose Department of Health has been implementing a Community Health Care Strategy since 2016 [24]. Among the actions that this strategy aims to carry out is the implementation of SPS in the 123 PHC Teams which reach out to a total of 1,326,261 people [29]. All PHC Teams in Aragon are direct public provision; healthcare professionals are mainly civil servants and use the same public EHR. Therefore, the assessment perspective is that of the public healthcare provider, the Aragones Public Health System. In order to develop SPS in a standardized way a Guide for Social Prescribing Schemes in Primary Health Care was created [9]. This guide describes the stages to follow when implementing SPS and instructions on how to register it in the patients’ EHR (Table 1).

**Table 1:** Protocol of social prescribing schemes in electronic health record of Aragon healthcare system

Protocol of Social Prescribing Schemes in Electronic Health Record of Aragon Healthcare System	
1st	Electronic Health Record Dashboard. To enter in patient’s EHR. Select a diagnosis to be linked to the social prescribing scheme.
2nd	EHR Protocol Access: To select the protocol: “PHC-Social Prescribing Scheme”.
3rd	Items to register in the patient’s SPS protocol: • To click on the skill or skills to enhance with the Social Prescribing Scheme (physical skills, self-care skills, cognitive skills, emotional skill, relational and social skills and others). • To write the reason for the recommendation.
4th	Website of Health Asset Finder (Health Assets validated by Aragon Public Health Authority) To search into and identify the Health Asset to prescribe.
5th	Health Asset To record the Health Asset prescribed.
6th	Official written information To give written information about the selected Health Asset (useful for the patient) and a document with information with full details of the SPS made (useful for the provider).
7th	Monitoring To organize an appointment to monitor the attendance to the recommended asset, the patient satisfaction and the improvement.

SPS can be indicated in patients’ clinical encounters by any PHC team worker (social workers, nurses, doctors, pediatricians, physiotherapists, midwives, among others).

The protocol establishes that SPS must be associated with a diagnosis and collects information about the aspects to be improved in the patient (physical activity, self-care, cognitive skills, emotional skills, relational and social skills, or others), the prescribed health asset and useful comments. Additionally, this protocol offers the possibility to refer the patient directly to social worker in case of specific social needs. The health assets are logged in the “Health Asset Finder of the Aragon Community Health Network” [30]. All health assets are registered in the finder by the provider that coordinates and facilitates them. All the health assets in the finder are validated by the Aragon Public Health Authority. The health assets may be provided by public administration, third sector or private entities.

Citizens with a SPS are monitored by PHC Teams. In this monitoring process, the protocol collects information about the number of times a patient attends the health asset, patient satisfaction and the degree of improvement perceived by the professional.

The SPS guide highlights the importance of coordination and cross-sectoral work for the implementation of SPS to avoid any possible iatrogenesis [31].

To develop an evaluation model of SPS in PHC, EvalRA study establishes two phases (Table 2).

**Table 2:** Phases of EvaLRA study

2021	2022	2023	2024
Mapping PHC Teams regarding Community Care Service. Selection of teams which implementing SPS within a patients’ encounters with SPS protocol in the electronic health record.	Consolidation and definition 1st set of indicators for the evaluation of SP with nominal groups and Delphi Survey.	Feasibility of the implementation of a set of indicators for SP Scheme evaluation in selected PHC Teams.	Sensitivity assessment, consolidation and definition of a set of indicators of structure, process and outcomes for social prescribing schemes in PHC.
PHASE 1		PHASE 2	

### Phase 1: Mapping and selection of participating PHC teams and the design of evaluation indicators. This will be achieved through

#### Analyzing the current situation regarding community health care services in the different PHC teams in Aragon

Currently, the Community Health Care Strategy is being followed by 123 PHC teams in Aragon, but not all teams are implementing it at the same degree.

The degree of implementation of the Community Health Care Strategy among PHC Teams will be measured using the PHC Team Community Health Agenda. The Community Health Agenda collects information about the community health group of each PHC Teams (the member of PHC teams working in community health). The agenda registers the activities and projects led by the community health group and other activities led by community entities in the same territory (educational centers, social centers, citizens’ associations, local institutions among others). The agenda also collects the creation of the health assets map and the implementation process of SPS.

According to the degree of development of Community Health Care Strategy, PHC Teams are classified in four levels:

Level 1: PHC Teams without community health group or community health care projects. Level 2: PHC Teams with a community health group and/or community health care projects [32]. Level 3: PHC Teams with a community health group and a community health agenda, carrying out a community health diagnosis and coordination with community entities in the territory [33]. And Level 4: PHC Teams implementing SPS within patients’ encounters and registration of SPS Protocols in the EHR [8].

In order to carry out this classification, a team of auditors will be trained to review the community health care services of each PHC Team.

#### The creation of a set of criteria and indicators to evaluate the implementation and impact of SPS in PHC teams.

In this phase, a SPS evaluation model will be agreed by experts and a first set of criteria and indicators will be defined. Therefore, the set will be submitted for their validation and monitoring in PHC teams that formally carry out SPS (Level 4). Providers and patients will also participate in the validation and monitoring process.

For the development of the criteria and indicators, a national and international scientific literature review will be carried out. Afterwards, two qualitative methodologies (nominal groups and Delphi survey) will be carried out to reach a model consensus.

Participants and procedure:

- Nominal Groups

The nominal group sessions will be carried out in the first year of the study.

There will be five nominal groups with 8-12 participants each. Participants will be from different regions of Spain. Two groups will be composed of PHC professionals, one with public health professionals and healthcare managers, one with health asset providers and one with patients. Each group will last 2 hours.

The criteria to be worked on the groups will be the relevance, effectiveness, efficiency, utility and sensitivity. It is expected that other criteria will appear during the sessions.

The results from the groups will be triangulated with consideration for the productivity of ideas, spontaneity and consistency.

These sessions will be held online through videoconferencing with the support of a software to facilitate the work during these sessions.

The sessions will be recorded with informed consent from all participants.

The information will be transcribed and analyzed, coding information regarding main community health process issues.

The information obtained in the group sessions will be used to create a set of criteria that will then be evaluated using the Delphi consensus survey.

- Delphi Survey

Approximately 120 people will be invited to participate in the Delphi survey. A minimum of 80 responses are expected to be provided.

Participants will be from different Spanish regions and belonging to different stakeholders in the process of social prescribing.

The Delphi survey will be sent through an online application and it is estimated that a minimum of two waves to reach consensus. The cut-off point will be determined according to the degree of initial consensus by using successive waves. The process will involve to discard those criteria with a very low degree of agreement and accept those with a high degree of agreement, using quartiles distribution. After the first wave, the participants will receive feedback on the results regarding the average scores of each criteria, as well as their self-scores and the inclusion of new proposals.

In both methodologies, all stakeholders involved in the implementation of SPS will participate: public health administrations, health managers, health asset providers, members of PHC teams, scientific researchers and citizens participating already in SPS.

The recruitment of participants for the development of both methodologies will be carried out using a snowball sampling method.

The inclusion criteria for participants will be the following: seven or more years of professional experience, participation in community health initiatives and experience with social prescribing. Equal representation of women and men shall be considered.

To finish this stage, a first set of indicators will be generated by the research group.

### Phase 2: The validation and monitoring of the criteria and the development of final indicators

The validation and monitoring of the agreed indicators will be carried out in the PHC teams classified as level 4 in terms of community health development. The selected PHC teams have performed a large number of SPS registered in the EHR of their territory patients list. The PHC teams will be audited to accomplish inclusion criteria. They will receive a brief training on the use of the indicators developed in phase 1 of the project.

For the validation process, a web application will be created to collect information about the feasibility and usability of indicators. The web application will be built up with a set of activity indicators from PHC teams and providers, and qualitative indicators to assess the usability from PCH teams, providers and patients participating in the SPS.

This information will be analyzed at the kick-off and after 6 and 12 months to search for sensitivity of changes in the indicators.

Once the information has been obtained, an analysis and validation of the results will be carried out considering current feasibility of the indicators (with the current health information systems available), their usability and sensitivity along the time. Finally, a synthetic index comparing PHC teams will be tested. As a result, the set of indicators will be consolidated to evaluate SPS in PHC.

A group of stakeholders involved in the SPS process (health asset providers, PHC teams, public health administration and healthcare managers and citizens) will identify and agree upon the necessary future changes in the health information system in PHC to cover up the set of indicators using the results from the validation process. This study may help to plan a broader development of a set of indicators in the future.

## Discussion

The complexity and novelty of social prescribing influences the variability and lack of evidence on several aspects related to it.

This study aims to develop a method to evaluate the application of formal schemes of social prescribing during routine care provided in Aragon PHC. Rather than using different criteria and indicators, this model proposes to develop a basic scorecard that may better assess the most important and viable aspects of the implementation of social prescribing.

The main limitation of this study is the short period of time during which the first assessments and measurements of the agreed indicators are to be made. More time and experience would be needed to be able to close the scorecard with certainty.

Likewise, the specific characteristics of the area where the indicators are to be piloted can be seen as a limitation, given the existing differences between health systems and social prescription models. However, this area can also be seen as a strength due to its history of implementing formal social prescribing schemes and the integration of this process within its electronic health record.

The results of the project will be provided to the Aragones health system managers so that they can be incorporated into the management agreements of the primary care teams in Aragon, standardizing and formalizing the evaluation of social prescribing. Likewise, at national level, the proposal will be forwarded so that it can serve as a model for evaluation in other regions. Internationally, this scorecard could be a model to be considered by other countries with similar healthcare structures to ours.

## Data Availability

Not applicable.

## Abbreviations

PHC: Primary Health Care
SP: Social Prescribing
SPS: Social Prescribing Schemes
EHR: Electronic Health Record
